# Supplementary material for: Lung Adenocarcinoma Diagnosed at a Younger Age Is Associated with Advanced Stage, Female Sex, and Ever-Smoker Status, in Patients Treated with Lung Resection
Source: Cancers (Basel). 2023 Apr 21;15(8):2395. doi: 10.3390/cancers15082395 (PMC10136510; doi:10.3390/cancers15082395)
Supplement: Supplementary file 1 [file cancers-15-02395-s001.zip › cancers-2223206-supplementary.pdf]

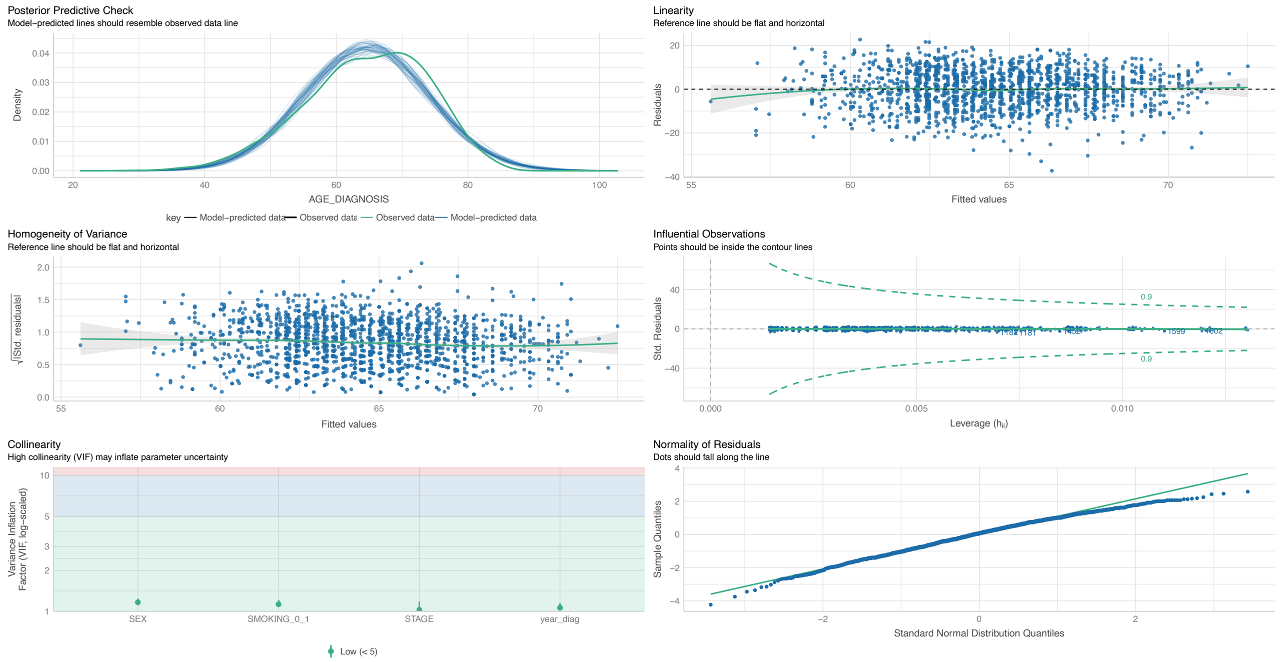

Supplementary Figure S1. Assessment of linear model assumptions in the discovery series with `check_model` function of Performance package in R. No multicollinearity was observed, but non-linear distribution of residuals and heteroscedasticity were detected.

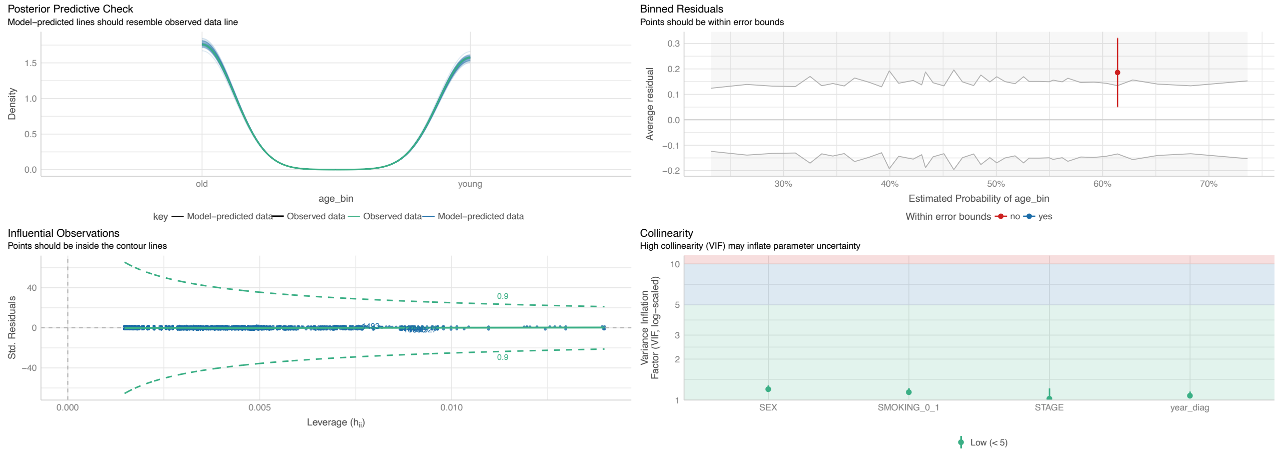

Supplementary Figure S2. Assessment of logistic model assumptions in the discovery series with `check_model` function of Performance package in R. No multicollinearity was observed; about 92% of the binned residuals are inside the error bounds. Additionally, no outliers were detected with Cook's method (threshold =0.89).

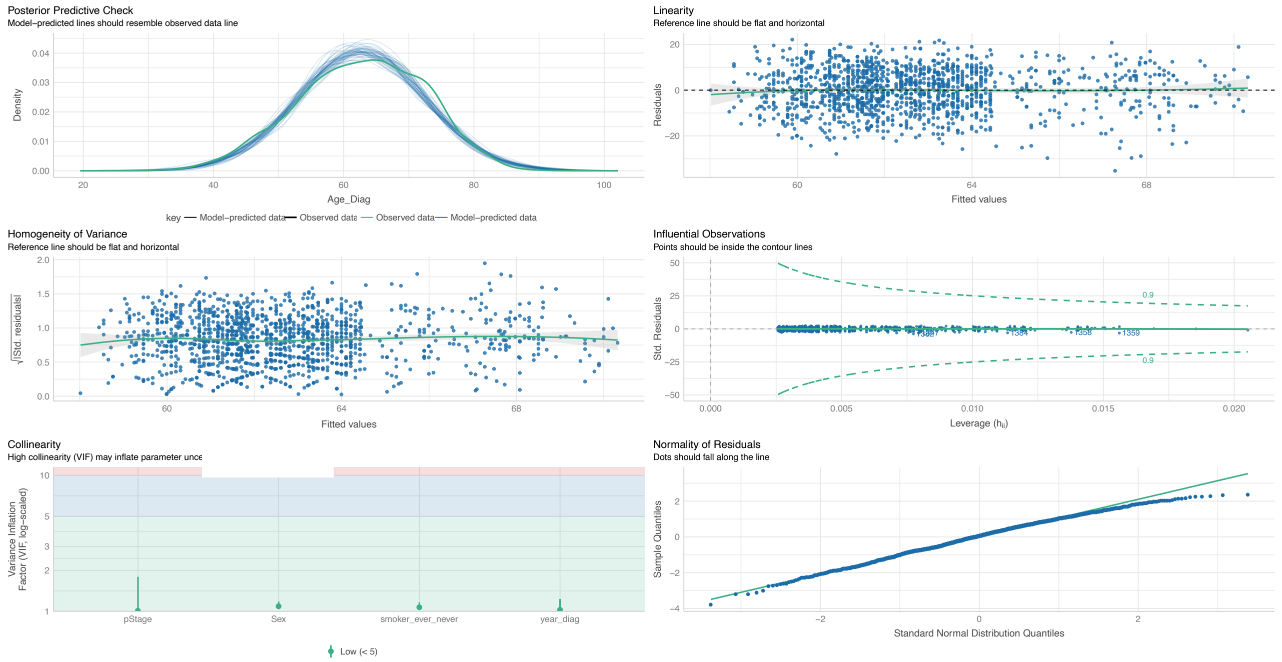

Supplementary Figure S3. Assessment of linear model assumptions in the validation series with `check_model` function of Performance package in R. No multicollinearity or heteroscedasticity was observed, but non-linear distribution of residuals was detected.

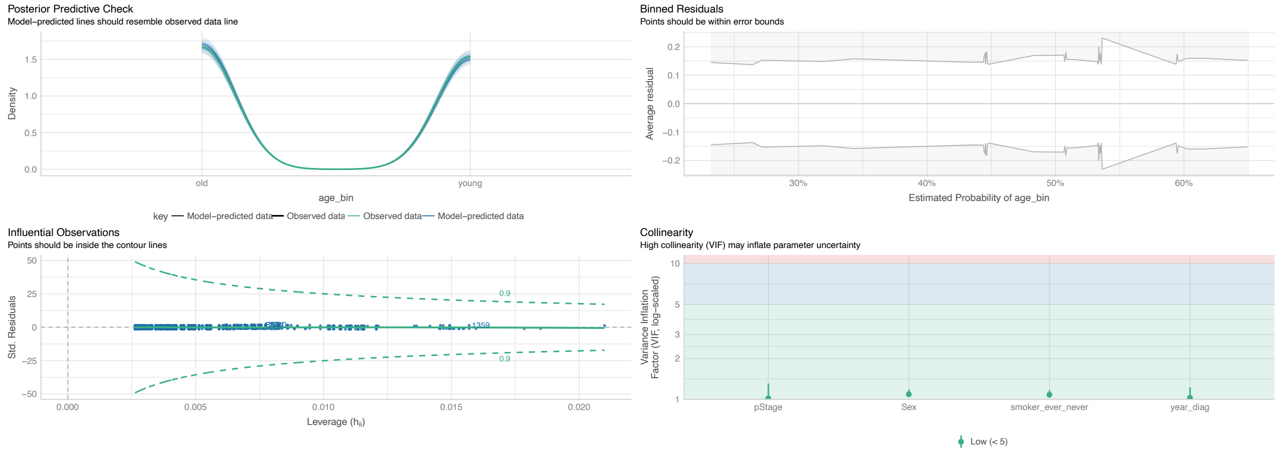

Supplementary Figure S4. Assessment of logistic model assumptions in the validation series with `check_model` function of Performance package in R. No multicollinearity was observed; about 100% of the binned residuals are inside the error bounds. Additionally, no outliers were detected with Cook's method (threshold =0.89).
